# Assessment of Biomarker Testing for Lung Cancer Screening Eligibility

**DOI:** 10.1001/jamanetworkopen.2020.0409

**Published:** 2020-03-05

**Authors:** Tricia L. Larose, Filip Meheus, Paul Brennan, Mattias Johansson, Hilary A. Robbins

**Affiliations:** 1 Genetic Epidemiology Group, International Agency for Research on Cancer, Lyon, France; 2 Department of Public Health and Nursing, Norwegian University of Science and Technology, Trondheim, Norway; 3 Prevention and Implementation Group, International Agency for Research on Cancer, Lyon, France

## Abstract

This exploratory study analyzes the cost-effectiveness of incorporating biomarkers into eligibility assessment for lung screening.

## Introduction

The National Lung Screening Trial (NLST) demonstrated that screening by low-dose computed tomography can reduce lung cancer mortality.^1^ However, benefits and harms depend on individual lung cancer risk, with benefit concentrated among high-risk individuals.^2^ Risk prediction models can identify high-risk individuals, but their performance is limited by reliance on easily assessable risk factors such as age and smoking history.^3,4^ Incorporating information from biomarkers could further improve risk assessment,^5^ allowing more deaths to be prevented. In this economic evaluation, we explored whether incorporating biomarkers into eligibility assessment for lung screening might be cost-effective.

## Methods

Our exploratory analysis assumed screening 9.0 million US ever smokers, the number eligible by US Preventive Services Task Force (USPSTF) criteria,^4^ with an NLST-like program (3 annual screens and follow-up). The reference scenario assumed smoking-model risk-based eligibility^4^ at no cost. We assumed that biomarker-informed eligibility would increase the percentage of future lung cancer cases classified as screening eligible.

We estimated the number of lung cancers in the reference scenario using the ratio of mean 5-year risks in the smoking model (4.5%)^4^ vs the NLST computed tomography group (2.79%; direct analysis). We repeated this for the USPSTF-eligible 9.0 million and all 43.4 million US ever smokers.^4^ We estimated false-positive screens as the mean between an upper bound, which fixed the proportion of true-positive screens among positive screens (0.036), and a lower bound, which fixed the proportion of false-positive screens among all screens (0.233).^1,2^ We classified 60.3% of cases as screen detected (true positives).^1,6^

We applied direct NLST costs using 2009 Medicare prices for screening (per person screened), workup (per positive screen), and treatment (per lung cancer case).^6^ For biomarker-informed eligibility, we assumed testing 50% of US ever smokers (21.7 million)^4^ with the biomarker test, as the precise number needing testing is unknown. We applied hypothetical per-person biomarker costs ranging from $5 to $300. For life-years gained, we used lifetime-horizon life expectancies for cases and noncases, with and without screening.^6^ Incremental cost-effectiveness ratios (ICERs) divided the additional cost of biomarker-informed eligibility by additional life-years gained, compared with the reference scenario.

We followed the Consolidated Health Economic Evaluation Reporting Standards (CHEERS) reporting guidelines for economic evaluations as applicable. This study does not constitute human subjects research and per Common Rule, no ethical review was required.

## Results

In the smoking-model reference scenario, assuming screening of 9.0 million ever smokers, 62% of the future lung cancer cases occurring among all 43.4 million US ever smokers were classified as screening eligible. The ICERs for biomarker-informed eligibility varied with the percentage of future cases classified as screening eligible and the per-person cost for biomarker testing (Figure).

**Figure.**
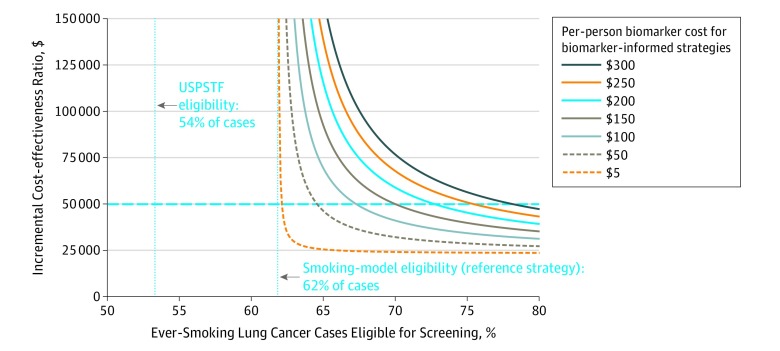
Incremental Cost-effectiveness Ratios for Biomarker-Informed Lung Screening Eligibility Based on Exploratory Cost-effectiveness Analysis The reference scenario defines eligibility using a risk prediction model incorporating smoking and demographic information. Incremental cost-effectiveness ratios (ICERs) are shown as a function of (1) the percentage of future ever-smoking lung cancer cases classified as screening-eligible and (2) the per-person biomarker testing cost. The x-axis shows the percentage of ever-smoking lung cancer cases classified as screening-eligible (54% based on the US Preventive Services Task Force [USPSTF] guidelines, 62% based on smoking-model risk-based eligibility, and increases up to 80% based on hypothetical biomarker-informed eligibility). Individual curves represent ICERs based on hypothetical per-person biomarker costs ranging from $5 to $300. The dotted horizontal line indicates an ICER willingness-to-pay threshold of $50 000 per life-year gained.

Assuming a willingness-to-pay threshold of $50 000 per life-year gained, biomarker-informed screening eligibility was cost-effective under some simulated scenarios but not others.

For example, if biomarker-informed eligibility increased the percentage of screening-eligible cases from 62% to 70%, then a biomarker costing up to $100 might be cost-effective (ICER = $41 721), whereas a $150 test might not (ICER = $50 910). If biomarker-informed eligibility made only a small improvement, from 62% to 65% of cases, then testing could cost at most $50 (ICER = $49 690). However, if biomarker-informed eligibility were able to capture 80% of future cases, then it could be cost-effective even at $300 per test (ICER = $47 289).

## Discussion

Our results suggest that using biomarkers to optimize selection of ever smokers into lung cancer screening may be cost-effective in some scenarios. We found that cost-effectiveness of biomarker-informed screening eligibility is associated with the degree to which it improves discrimination as well as the biomarker cost. Although the results from this exploratory analysis do not provide sufficient evidence for price setting or decision-making, they do support the pursuit of additional research on biomarker-informed eligibility for lung cancer screening.
